# Vaccination in the Light of the Royal British Commission

**Published:** 1898-10

**Authors:** M. R. Leverson

**Affiliations:** Fort Hamilton, L. I., N. Y.


### VACCINATION IN THE LIGHT OF THE ROYAL BRITISH COMMISSION.

M. R. Leverson, M. D., Fort Hamilton, L. I., N. Y.

(Continued from page 344.)

When we reflect that it is precisely this "glycerinated lymph" which the British Local Government Board is now (June, 1898) trying to get Parliament to force upon the people of Great Britain by a bill introduced by the President of the Board (Mr. Chaplin), and which passed its second reading in the House of Commons on May 9th, what criticism should be passed upon such conduct? The contention of the Local Government Board, made on June 11th, 1890, by their mouthpiece, Lord Herschel, the Chairman of the Commission, was (Q. 9,798) that vaccination with this glycerinated lymph "was not vaccination proper, in our sense of the term, because it was not simply the insertion of vaccine matter, as there were foreign matters used, while the German Commission's suggestion is that it was in those foreign matters that the mischief existed."

At Q. 9,835 Mr. Tebb takes up the disaster to forty children at Villefranche-D'Aveyron. This was communicated to a public meeting held at Leicester on March 23d, 1885, in a letter from Dr. Charles Pigeon, of Forchambault. Of forty children there vaccinated nine died within forty-eight hours. This case is remarkable for the direct and positive falsehood by the Mayor of Villefranche (one Andrieux), who, in reply to Mr. Tebb's inquiry, wrote to him on April 16th, 1885, absolutely denying that anything of the kind had taken place, and that the entire story is "nothing but a culpable invention of the reporters." Mr. Tebb also showed that up to May 1st, 1885, in response to an inquiry in Parliament by Mr. C. H. Hopwood, the British government professed to be ignorant of the calamity. The whole story came out afterwards in an official report (Q. 9,840).

Q. 9,844. "On March 13th, 1885, Dr. Andrieux, of Asprierès, vaccinated forty-two infants. All were attacked with fever, and on the following day six were dead. Their bodies were covered with black patches."

In the history of the cases and of the sources of the vaccine, it is to be observed that some of the children vaccinated with material from the child from whom the chief vaccinifer was vaccinated did not suffer at all. In the case of this vaccinifer (the child Clapier), the disease ran a more rapid course than usual, as it did also in the case of another child (Bessons) vaccinated at the same time; otherwise neither the vaccinifer nor Bessons suffered. Even the children vaccinated from Clapier who re-recovered suffered severely, and, as among other things, they suffered from impetigo, it is fairly to be questioned whether they have not been rendered liable to cutaneous disorders for the rest of their lives.

At Qs. 9,856 to 9,860 Professor Michael Foster harks back to the Rütgen disaster to try and blame the glycerine (which the British Local Government Board now desires to make general in England) instead of the vaccine poison; but Mr. Tebb answers: "My point in all these cases is, What satisfaction can any explanation afford to the afflicted victims? If children are injured, or suffer for life, or are killed, what satisfaction can it be to have it shown that the misadventure is due to diluted or to spurious vaccine, or to vaccination where there is an epidemic of disease, or vaccine from one institute or another, or the carelessness of the operator? My contention is that these explanations are all extremely unsatisfactory from a public point of view, and (Qs. 9,864–6) the Commissioners entirely acquit the physicians."

On June 18th, 1890, Mr. Tebb resumed his testimony.

Q. 9,954 proposes to deal with the Denkschrift or memorandum of the Board of Health of Berlin presented by the German Government to the Imperial Vaccination Commission of 1884.

Q. 9,955. Sets out a letter to Prince Bismarck from the London Society for the Abolition of Compulsory Vaccination, in which, after reciting the various contentions of Jenner, the society asks that the Commission be directed to define the proper virus commencing with Jenner's horse-pox—cow-pox, proceeding to cow-pox and then to horse-pox, and lastly to small-pox—cow-pox; and consider how these various forms of virus are affected by transmissions from child to child and animal to animal; and how the advocates of each of these varieties of virus attest their efficacy against small-pox.

Q. 9,557. The Lebus disaster is reported in the Appendix to the official report of the German Vaccination Commission of 1884. Also there appears a memorandum dated Berlin, March 28th, 1885, "At the time when the vaccination law was promulgated the opinion prevailed generally that the dangers connected with vaccination to the life and health of the patient were unimportant or rather, did not exist at all. Thus in No. 4 of the final conclusions of the opinion drawn up by the Royal Russian Scientific Deputation for medical affairs dated February 28th, 1872, which document formed the principal basis for the projected law, "That there existed no warranted fact in favor of a deleterious influence of vaccination upon the health." It was, however, seen subsequently that this thesis could not be upheld. In fact, very serious damage by vaccination has occurred anything but rarely, both before and after the promulgation of the vaccination laws. . . . Up to the year 1880 fifty cases have become known in which syphilis inoculated with the vaccine caused illness to about 750 persons (Lotz on *Small-pox and Vaccination*, 1880, p. 13)."

Q. 9,958–60. This is given by the Berlin Board of Health. It is a memorandum prepared in the office of the Berlin Board of Health. It appears as an Appendix to the report of the German Commission, p. 359, No. 287. [The Berlin Board of Health adopt the statements quoted from Lotz as full authority. —M. R. L.]

Q. 9,961. Continuing to quote, Mr. Tebb reads from this report of the German Commission, p. 359, No. 287: "A few separate cases of vaccine syphilis may, perhaps, be looked upon as being uncertain; but, on the other hand, others were not made publicly known, so that the figures quoted above are likely to be less than the number of cases that happened in reality.

Still greater dangers than those connected with vaccine syphilis are threatened by vaccine erysipelas, which, as is now generally admitted, are far from uncommon. . . . A number of cases of general illness taking place *en masse* have been registered which happened immediately after vaccination, and in accordance with the latest experience, derived from the etiology of erysipelas, admit of no other explanation beyond their having been caused by vaccination direct." "Other diseases, also, have been transmitted by vaccination, or, at least, the possibility of such transmission must be admitted." Thus it is possible that septic processes of disease, belonging to the class of wound infection diseases, can be caused by vaccination, as is proved by the fact of the inoculated persons at Grabuist falling ill *en masse*. Some observations made on the origin of sores and inflammations of the lower cellular tissues of the skin after vaccination must also be included in this class. The transmission of tuberculosis and scrofula by vaccination are then referred to as not capable of being contested, though probably not possible of positive proof, because "the first symptoms of these diseases occur too late after infection to admit of an incontestible connection between infection and visible disease being ascertained." The report then says: "In view of these experiences it is impossible any longer to represent vaccination as was done at the time the vaccination law was under discussion, as being absolutely free from danger to the health of the vaccinated person." The report further states: "In the first place, vaccine syphilis will always have to be feared, which forms also the principal arm in the hands of the opponents to vaccination. It has certainly been stated that all cases of vaccine syphilis can be traced back to the negligence of the vaccinating surgeons, and that under due observance of all precautionary measures, vaccine syphilis can be avoided." This is shown to be erroneous, and the report then gives the case at Lebus, in 1876, of vaccine syphilis in fifteen revaccinated school girls. "The lymph had been taken from a child seven months old, which at the time of vaccination appeared to be perfectly healthy, and *was found to be healthy on the occasion of various* examinations made later on. . . . On the occasion of a judicial examination the mother was found to be free from syphilis."* In 1879 the Petitioners' Commission of the Reichstag "proposed that inquiries should be instituted as to the present extension of infantile syphilis."

Q. 9,963. Mr. Tebb's next point was with regard to five cases of vaccinal syphilis in the Department de l'Oise, France, reported August 6th, 1889, before the Academy of Medicine. Communicated by M. Hervieux. "David Marie Chapins, aged thirty-two years, living at 105 Rue de Sevres, presented herself, July 27th, 1889, at the Hospital of St. Louis, with her child, born November 28th, 1888, and therefore aged eight months. Of good constitution; never ill. Vaccinated at the Academy the 11th of last May by five punctures on each arm. He had five vesicles on one side, four on the other, which developed normally and are perfectly healed. Brought back after eight days that the certificate might be obtained, this child was utilized for the collection of vaccine; some tubes and some glasses were charged. A month later one of the vesicles, that from which the vaccine had been taken, broke, and it presents now, under the form of an erosion, slightly swollen, a gray or red surface, with red-colored surroundings, inflamed by rubbing (the child's arm had not been dressed), the base like parchment, with corresponding axillary ganglion. In the neighborhood four or five papula, clearly specific. Rash scattered over the body. Syphilides on the soles of the feet and palms of the hands appeared in erythematous form. Nothing at the mouth. One dry papule under the scrotum." Two days later another similar case, both diagnosed by himself and Dr. Fournier as syphilis vaccinale, both children otherwise in excellent health. In the case of young children acquired syphilis is seldom as serious as hereditary syphilis. "The erosion on the shoulder of each child was transformed to

---

* That is to say, syphilis developed in the vaccinees, although the vaccinifer was wholly free from the infection. In the cases of these unhappy school girls, for some cause or other, the cow-pox or horse-pox, which doubtless served as the original source of inoculating material, *reverted* to its original malignancy. It was a case of simple atavism, whereof biology furnishes so many instances, and what the original character of cow-pox is was shown in the pathological table, *supra*, pp. 241–244.

a thick yellow crust." Dr. Weill offered to assist M. Hervieux in the painful task of investigation. They found the vaccinifers in excellent health. The more robust of the two, a boy, has never been ill. The mothers were examined by M. Fournier and M. Weill and found to be "*irreproachable*." The vaccinifers were produced before the Academy. Their investigation led to the discovery of other cases of vaccinal syphilis. One case, a boy of five months, vaccinated at the Academy May 11th last, "ulceration of a bright red under one of the arms, erythematous syphilides on the trunk, level with the vaccination scars on the limbs, on the genitals, and the soles of the feet." Another case was a girl of two and one-half years. "Specific ulceration of the upper part of the left arm, erythematous spots on the trunk and in the region of the anus." Another case is a child of four months, presenting two specific ulcerations on the shoulder and dry, scattered erythemateuse. Also two who have died since the vaccination of May 11th. One was a pale, puny child from birth, but no trace of specific disease; the other was carried off by diarrhœa and convulsions. "Out of fifty-three children vaccinated May 11th, 1889, there have been five children rendered syphilitic; two have died of diarrhœa and convulsions, but not syphilitic; six children whose parents have given a false address; thirty-nine children visited and proved free from any ill effects, and one sent out to nurse reported in good health." And the commission concludes from the facts that "M. Hervieux has had the misfortune to come upon a child in a state of latent syphilis!" "It is a danger which is, and always will be, impossible to avoid," and goes on to recommend animal vaccine. He also mentions eleven previous cases of vaccinal syphilis in 1865, and concludes, "It is too much! We see human vaccine has demonstrated what it is; let us not give it time to realize a third catastrophe."

[As to what animal vaccine is, see pathological table in The Homœopathic Physician for June, 1898, Vol. XVIII, pp. 241–244.]

